# Patient-derived xenograft (PDX) models, applications and challenges in cancer research

**DOI:** 10.1186/s12967-022-03405-8

**Published:** 2022-05-10

**Authors:** Shahrokh Abdolahi, Zeinab Ghazvinian, Samad Muhammadnejad, Mahshid Saleh, Hamid Asadzadeh Aghdaei, Kaveh Baghaei

**Affiliations:** 1 Basic and Molecular Epidemiology of Gastrointestinal Disorders Research Center, Research Institute for Gastroenterology and Liver Diseases, Shahid Beheshti University of Medical Sciences, Tehran, Iran; 2 Department of Applied Cell Sciences, School of Advanced Technologies in Medicine, Tehran University of Medical Sciences, Tehran, Iran; 3 Cell-Based Therapies Research Center, Digestive Diseases Research Institute, Tehran University of Medical Sciences, Tehran, Iran

**Keywords:** Cancer animal model, PDX, Preclinical study, Humanized model, Avatar model of cancer

## Abstract

The establishing of the first cancer models created a new perspective on the identification and evaluation of new anti-cancer therapies in preclinical studies. Patient-derived xenograft models are created by tumor tissue engraftment. These models accurately represent the biology and heterogeneity of different cancers and recapitulate tumor microenvironment. These features have made it a reliable model along with the development of humanized models. Therefore, they are used in many studies, such as the development of anti-cancer drugs, co-clinical trials, personalized medicine, immunotherapy, and PDX biobanks. This review summarizes patient-derived xenograft models development procedures, drug development applications in various cancers, challenges and limitations.

## Background

**Fig. 1:**
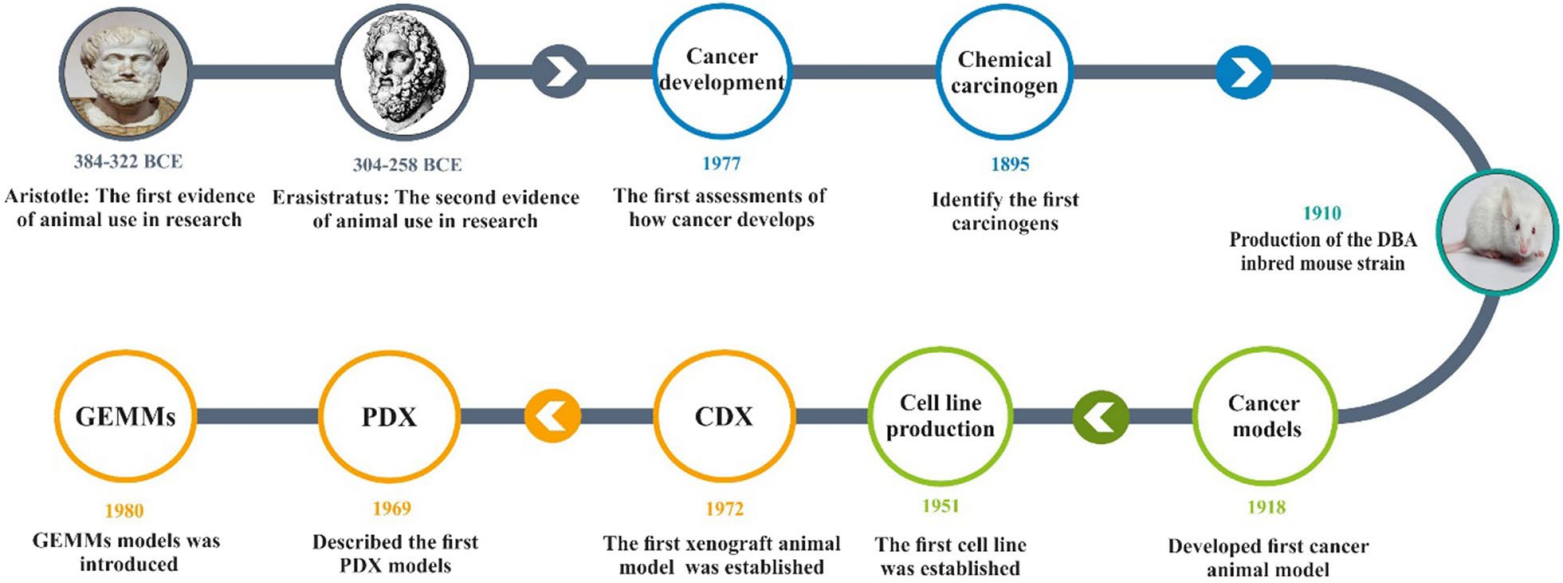
Cancer animal models timeline. The timeline shows the first available reports of the use of animal models during the Aristotle and Erasistratus eras, which over time, recognizing how cancer formed in 1777 and identifying carcinogens paved the way for the use of cancerous animal models in 1910 with the development of DBA mice. Finally, in 1918, the first cancer model was developed by Yamagiwa and Ichikawa. Following the development of the first cell line in 1951, the first CDX model was introduced, which greatly contributed to the improvement of cancer science. Furthermore, PDX and GEMMs were introduced in 1969 and 1981, respectively. *GEMMs* genetically engineered mouse models, *PDX* patient-derived xenograft, *CDX* Cell Line-Derived Xenograft model, *DBA* dilute, brown and non-agouti

The first sparks of cancer models were formed more than 200 years ago when the first report of cancer by environmental factors was presented [1]. In recent century, mice have been widely used in biological research and have made significant contributions to cancer discoveries as a bedrock for cancer models [2] (Fig. 1). Evaluation and classification of cancer models gave scientists a relatively deep insight into the underlying genetic mechanisms of malignancy and cancer progression, and animal models with clinically predictive properties switch candidate drugs to phase 2 clinical trials with confidence from the pre-clinical phase. Therefore, cancer animal model studies focus on the pre-clinical evaluation of the efficacy of biological and chemical agents. However, more than 90% of drugs that successfully pass preclinical studies are ineffective in the human phases. These data suggest that conventional preclinical models such as monolayer cell lines culture or syngeneic and xenograft models are the main reason for the failure of most anti-cancer agents in humans [3, 4].

Validation of disease model to achieve the best model is essential; three criteria are usually evaluated. Face validity is the first criteria that describe the biology similarity between the human disease and the animal model. The second is target validity which the target agents should have a similar function in the model as in the clinical aspect, and the third is predictive validity which demonstrates that clinically effective therapeutic agents show a similar effect in the disease model [5].

Hence conventional models such as tumor cell lines showed different phenotypes after adaptation to in vitro culture conditions that were frequently distinct between laboratories, thus having a low resemblance to parental tumors. Indeed long-term in vitro cell culture showed alterations in tumor hallmarks caused by epigenetic and genetic changes. Therefore, along with the advantages, these significant limitations lead to low scores of these traditional models from the evaluation criteria of animal models and prevent using these models for drug screening and estimating the pre-clinical efficacy of drugs [6] (Table 1).

**Table 1:** Advantages and limitations of different animal models

Animal models	Advantages	Limitation	Ref.
Chemical carcinogenesis	Simplicity Assess the cancer process from initiation to metastasis in order Gene analysis in different stages	Not cover all cancers Tumor rejection by host immune cell Mice lifespan isn't enough for tumor induction Concerns about long-term use of carcinogens	[117, 118]
Syngeneic mouse models	Immunocompetence Simplicity High engraftment rate	Non-synonymous mutations Lack of heterogeneity The limited number of cell lines	[119]
GEMMs	Evaluating drug responses, resistance, and toxicity Allows to answer unique biological questions	Interspecies differences Random transgenesis Genetic compensation Lethality of some mutations Complexity of disease	[120]
Cell Line-Derived Xenograft model	Suitable for mechanism studies Rapid growth Evaluate non-targeted cytotoxic agents Available and cheap	Lack of heterogeneity Lack of immunological agents Lack of tumor micro-environmental	[77, 121]
PDX models	Retain heterogeneity and mutations Tumor microenvironment Intact endocrine system Metastasis assessment Tumor biobank formation	Generated in mouse with deficient immunity Different take rates Not suitable for early-stage cancer	[122, 123]
Humanized mice	Correctly mimics human tumor microenvironment Predictors of drug response in human cancer Creates a natural heterogeneity of tumor cells	Expensive technically complicated	[33, 124]

*GEMMs* genetically engineered mouse models, *PDX* patient-derived xenograft, *CDX* Cell Line-Derived Xenograft model)

Stringent success criteria during the preclinical stages have improved failure rates of the drug development process. The selection of animal models affects the success rate of these stages, which allows for evaluation of the target validity, which can predict the clinical efficacy of specific agents. As a result, the expectation is not to reproduce the human disease with all its complexities in the animal; instead, the model evaluates particular aspects of the disease. When using an animal model, it is crucial to ensure that the chosen model is fit-for-purpose.

In recent years, some aspects of animal models have been modified to improve the translational value. One of these approaches is to create humanized mouse models. These models are engrafted with human cells and tissues that impart human characteristics to mice and make the model a valuable tool for clinical translation [7]. The humanized mouse models mimic the human immune system; therefore, these models are mainly used to create patient-derived xenograft (PDX) models for cancer studies. In this different generation of animal models, the cancer patient's tissue is transplanted into immunodeficient mice. In subsequent years, studies have evaluated the accuracy of these models in responding to chemotherapy compared to the patient with donated tumor tissue; in most cases, there was a significant correlation between the response to chemotherapy in PDX models and patients [8]. Moreover, PDX evaluation showed significant heterogeneity and faithfully recapitulated the characteristics of their parental tumors on the microscopic, genetic and functional levels [9] (Table 1).

But the increased use of PDX models was postponed until The National Cancer Institute stopped using the (NCI)-60 panel (2016) and introduced the PDX model as a more reliable model [10]. Then it has become popular due to recapitulation clinically relevant. This review intends to further evaluate the PDX model as an applicable and reliable animal model.

## PDX models present parental tumor microenvironment structure

**Fig. 2:**
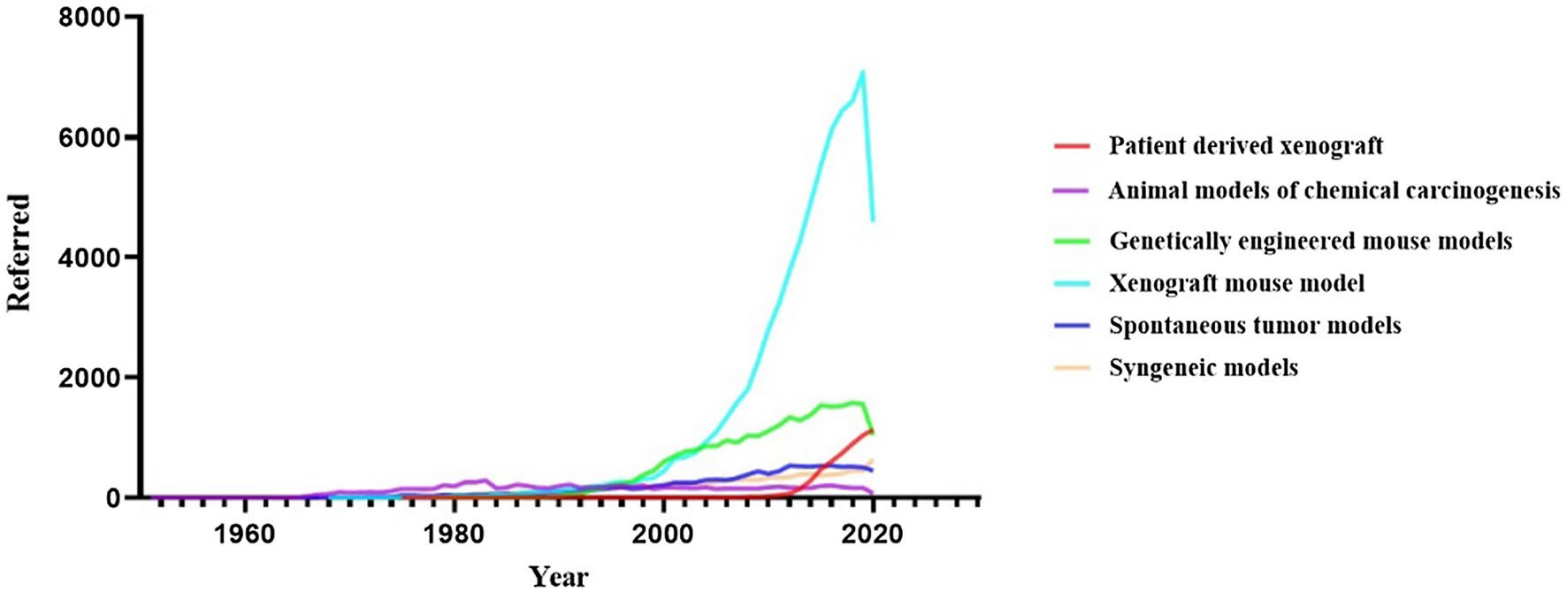
Cancer animal models over the years. The animal model of cancer was first introduced in 1918, but the beginning of today’s models was in 1951 with the development of the CDX models, but the development of other models led to competition for a more suitable model with more efficiency. The beginning of the twenty-first century can be considered the beginning of the flourishing of animal models of cancer. CDX model won the competition between the CDX and GEMME models because of its availability and is still the first choice in many studies. But the remarkable thing is that at the beginning of the second decade of the twenty-first century, PDX models have attracted attention and are being used in various studies with considerable speed. Data obtained from the PubMed database. *GEMMs* genetically engineered mouse models, *PDX* patient-derived xenograft, *CDX* Cell Line-Derived Xenograft model)

In the evaluation of cancer, the surrounding microenvironments should be considered. This microenvironment includes stromal cells, which are composed of Tumor Endothelial Cells (TECs) and Cancer-Associated Fibroblasts (CAFs), Tumor-Associated Macrophages (TAMs) that have a significant effect on cancer progression and metastasis [11, 12]. These cells produce Extra Cellular Matrix (ECM), a network of proteoglycans such as laminin, collagen, and fibronectin that regulates cellular polarization, intracellular signaling and, migration, and creating flexible, stable, and supportive structures for different tissues [13, 14]. ECM compounds are essential in the distribution of drugs in tumor tissue. While the cancer cell lines commonly used to create animal models do not have apparent clinical features of the primary tumor, PDXs are preferred because of preserving tumor microenvironment’s structure. Although the PDX models have some limitations, but its features have primarily eliminated the shortcomings of other cancerous animal models. High tumor heterogeneity, maintaining tumor-stromal, gene expression, and tumor tissue mutations and high predictive value make this model ideal for biomarker evaluation, evaluating cell-based therapies, pre-clinical studies, and use for personalized medicine [8, 15, 16]. These features have made it more popular in recent years and, unlike other models in the Covid-19 pandemic era, have not declined (Fig. 2).

### Methodology to establish PDX models

Generally, these models can be created as orthotopic, heterotopic, and metastatic. In heterotopic models, at the first step, the tumor tissue specimen should be prepared in a cold solution containing Fetal Bovine Serum (FBS), penicillin/streptomycin to increase the success rate of engrafting. The tumor tissue can be transplanted in three approaches; the tumor tissue can be cut into pieces and several 3–5 mm^3^ pieces, a large amount of tumor or injection of minced tumors can be used for transplantation. The tumor samples are washed three times with the above-mentioned solution before transplantation. Various parts of the mouse can be used as tumor tissue transplantation sites to create PDX models. The tissues can be engrafted heterotopically into the intracapsular fat pad [17, 18], the anterior compartment of the eye [19], under the renal capsule [20], subcutaneously, and orthotopically into the origin of cancerous tissue [21]. Orthotopic transplantation is usually preferred because it has a closer microenvironment to human cancer. But in general, both subcutaneous and orthotopic sites are most commonly used [19].

**Fig. 3:**
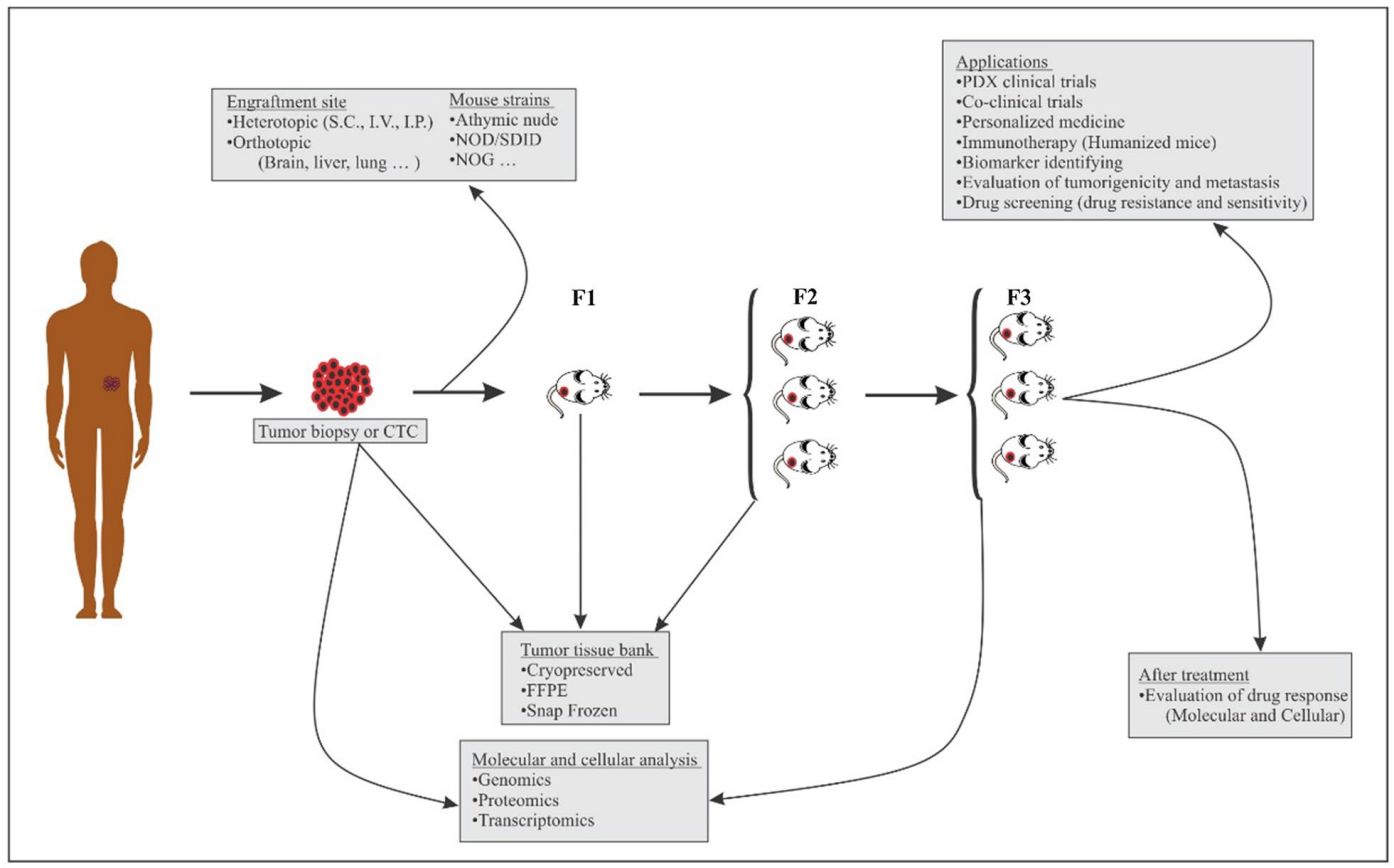
The process of creating PDX models and their applications. The generations are named F1, F2, F3, etc. Different cancer tissues can be used to create PDX models (orthotopic or heterotopic engraftment), as well as different hosts with varying degrees of immune deficiency. Tumor biopsy and generations F1 and F2 can be sampled for tissue bank, and tumor biopsy and F3 can be used for Genomics, Proteomics, and Transcriptomics analyzes. The F3 generation of these models can be used in various studies. *CTC* circulating tumor cell, *S.C* subcutaneously, *I.V* intravenous, *I.P* intraperitoneal, *FFPE* formalin-fixed paraffin-embedded

It should be noted that depending on the type of tumor and the sample area, the concentration and type of antibiotics may change. Small incisions are created on the lower back for subcutaneous transplantation in 4–8-week-old immunodeficient mice, and the tumor tissue sample is placed in each of the surgical areas. The remaining tumor tissues can be stored (− 80 °C for short term and in a liquid nitrogen freezer for long term storage) for genomic and protein evaluations compared with the xenograft tumor. The first generation F1and the subsequent generations are named F2, F3. Although designation can be done in other ways such as (G1, and so on) [22–24]. Reports suggest that the efficiency of nu/nu athymic mice is 75% and that NOD/SCID mice are more commonly used in F1 [25, 26]. The creation of NOD/SCID/IL2rγnull (NSG) mice resulted in higher efficacy of 95–100% in tumors that are difficult to transplant [27] (Fig. 3).

Xenograft tumors are assessed at least twice a week by a vernier caliper to measure tumor length and width. The time required to reach cancer depends on type of cancer, the location of the transplant, and the recipient strain. It takes an average of 2–4 months, and the transplant has not been successful if no tumor is detected over 6 months. When the size of the tumors reaches 1–2 cm^3^, we can begin the tumor passage. Finally, the third generation (F3 or G3) can be used for drug treatment and commonly employed in studies. However, genetics and histology (rather than merely the number of passages) should be the main determinants of PDX derivation from the patient’s tumor [28–30].

In recent years, the development of PDX models in humanized mice has been considered, in particular form in humanized PDX models, human immune system and human tumor tissue both together will be engrafted to the model and then human immune system reconstitute in an immunodeficient mice along with patient tumor engraftment [31].

There are three main classes of humanized mice; (A) human gene transgenic mice that are modified by specific human gene expression; (B) humanized organ mice which in this model the mice carrying a specific organ of human such as human hepatocyte infusion; (C) humanized immune system mice which are established in immunodeficient mice and human cells will reconstitute the mice immune system which describe briefly in Table 2 [32, 33].

**Table 2:** Establishment of humanized immune system mouse models

Humanized establishment method	Mouse strain	Rout of administration	Advantage	Limitation
PBMC engraftment	NOD-SCID mice	Intravenous injection of PBMC (5–10 × 10^6^), the engraftment consists of T cells	Cost effective, simple establishment pattern suitable model for T-cell-related immune research	Lack of necessary cytokines in order to B and NK *cell *in vivo proliferation, GVHD development makes a short period for experiment
Human HSC engraftment (CD34+) from BM, UCB, FL, MBP	NOD-SCID, NSG	Intravenous injection of 1 × 10^5^ HSCs, when the count of human CD45+ > 25% in peripheral blood the model is established	More complete immune reconstitution, GVHD rarely occurs	Long period of model establishment, maturation of human T cells in murine thymus makes human T cell restricted to mouse H2
Human BLT (bone marrow, liver, thymus) model	NOD-SCID, NSG	Intravenous injection of CD34+ HSC (0.5–1 × 10^6^) from human bone marrow, implantation of human fetal liver and thymus in to mouse sub renal capsule when the count of humanCD45+ > 25% in peripheral blood the model is established	Maturation of T cells in autologous human thymus, human T cell restricted to human HLA, highest immune reconstitution; B, T, macrophages and dendritic cells. long term maintenance of model	GVHD development due to mouse DCs, positive and negative selection processes of human T cells; although lighter GVHD than PBMC humanized model. Engraftments should carry from same donor, complex technique and ethical problems

## Applications of PDX models in cancer research

### PDX clinical trial

**Fig. 4:**
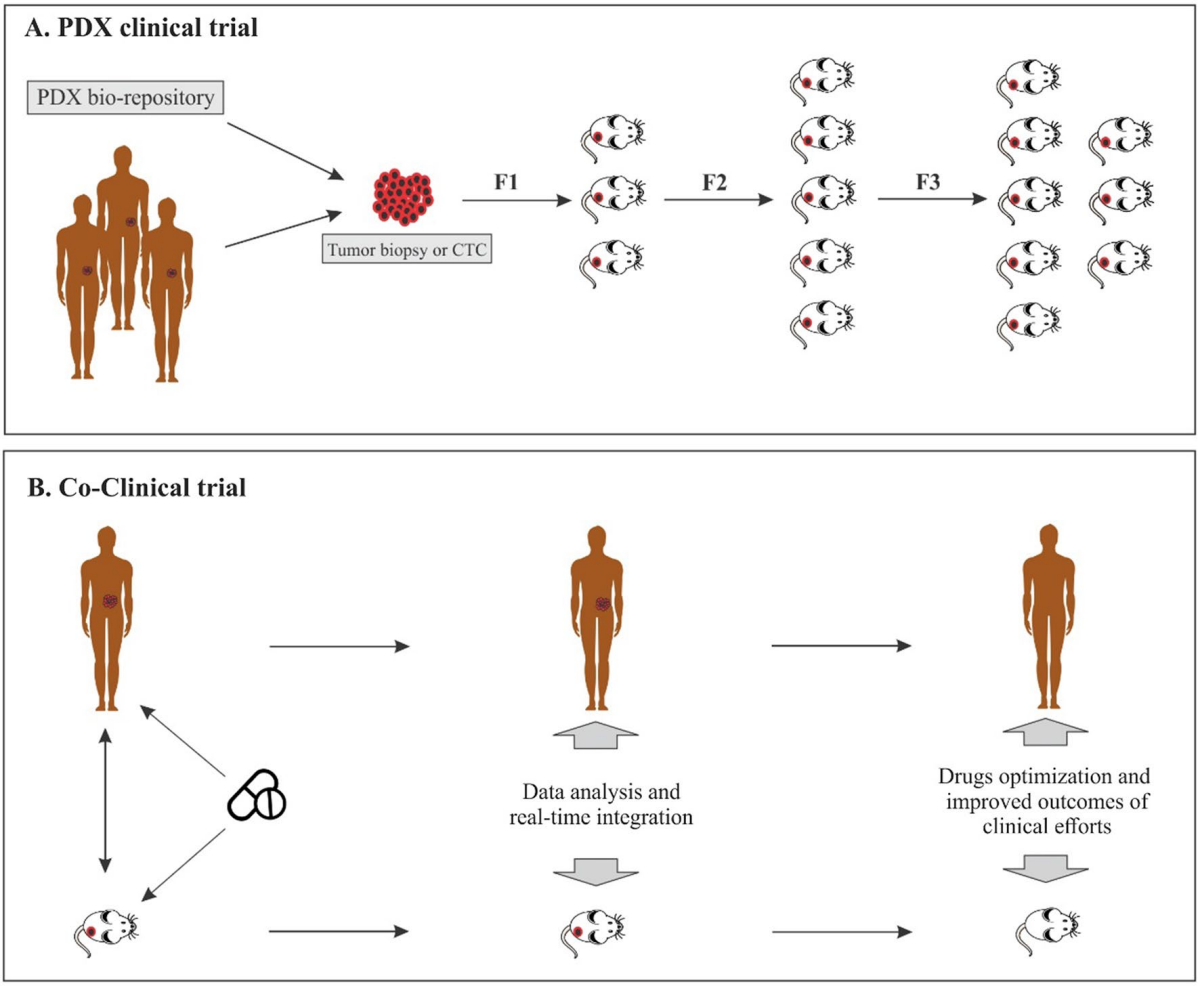
PDX clinical trial and Co-clinical trial. **A** In models that follow the PDX clinical trial approach, a large number of PDXs originate from several patients or samples of a bio-repository. Each model tests a specific drug regimen, and the information obtained is evaluated. **B** Co-clinical trials were developed to achieve precision medicine. In fact, the mouse trials are performed in parallel with the human trials, and then in real-time, the information obtained from the mouse study is transferred to the human study and integrated. This leads to the most effective clinical outcome

PDX Clinical Trials (PCTs) are substantial for clinical decision-making before human clinical trials and the development of anti-cancer agents. PCT is referred to as “phase II type clinical trial-like models”. In 2015, Gao et al. designed a high-throughput in vivo drug screening method, “1 × 1 × 1”, which intended one animal per model per treatment using a large number of PDX models. However, two or three animals per model per treatment (2 × 1 × 1 or 3 × 1 × 1) PDX clinical trials because of more representative of generalizable drug response have recently become more common [34] (Fig. 4A).

PDX models have become a powerful tool for evaluating drug efficacy and drug sensitivity, also known as PDX clinical trials. As shown in Table 3, several studies have tested the response rate of different drugs to different cancers in PDX models. These studies have shown that the response rate of PDX models to the drug is correlated with clinical outcomes [35]. In fact, before a human clinical trial, PDX clinical trials are critical to evaluate anti-cancer therapies. Accordingly, these cancer model features have led research centers and pharmaceutical companies to develop PDX repositories. The Novartis Institute of Biomedical Research has launched the PDX library. In this project, Novartis has created more than 1000 Avatar mice of various cancerous tissues, technically, there is no difference between Avatar and PDX, but an Avatar is mainly used in cases where the goal is personalized medicine; they use the predictive capacity of this model for particular patients, in contrast, PDXs are more commonly used in preclinical studies of drug evaluation and co-clinical trials. Accordingly, research centers and large pharmaceutical companies seek to establish a PDX repository, to be used in the development of preclinical studies on anti-cancer drugs [36]. Europeans have established a consortium called EurOPDX to store PDXs, which currently holds more than 1500 PDXs samples [37, 38]. Since 2017, the NCI has also been developing a national repository of Patient-Derived Models (PDMs), including PDX models. The overall goal of NCI is to establish a long-term storage site for at least 1000 PDX models so that researchers have sufficient biological and clinical diversity to conduct their studies [39, 40].

**Table 3:** Cancer PDX models in therapeutic approaches

Tumor histotype	PDX model	Treatment/molecular alterations	Response rate (RR)	Ref.
Breast cancer	Orthotopic	Docetaxel, 5-fluorouracil, trastuzumab	71%	[125]
		Docetaxel, doxorubicin, trastuzumab + Lap	100%	[126]
Colorectal cancer	Heterotopic	Cetuximab, panitumumab	100%	[80]
	Heterotopic	WT KRAS	100% responded to cetuximab	[127]
	Heterotopic	Oxaliplatin	92%	[79]
Ovarian cancer	Heterotopic	Cisplatin	81%	[128]
	Heterotopic	Doxorubicin, cyclophosphamide, 5-fu	0–27%	[42]
Gastric cancer	Heterotopic	Regorafenib	96%	[129]
Non-small cell lung cancer	Heterotopic	EGFR mutated	10% responded to gefitinib	[130]
Pancreatic ductal adenocarcinoma (PDAC)	Heterotopic	Gemcitabine	17%	[131]

### Cancer biology

This model has helped increase our understanding of the response of tumor cells to drugs, which leads to effective treatment strategies. Other applications of such models include creating PDX resistance to treatment, in which the consecutive administration of a particular drug can lead to the production of drug-resistant PDX models. These resistant tumors are more consistent with cancerous cells than cell lines, which can be used to trace drug resistance biomarkers and investigate drug resistance mechanisms [41]. For example, ovarian cancer exposed to long-term cisplatin induces resistance to this substance, Similar to clinical conditions. These models are used to find new anti-cancer agents, to evaluate suitable drugs for patients with platinum resistance [42]. Intratumoral heterogeneity of primary tumors in patients is recapitulated by PDX tumors, increasing evidence indicating that tumors have a distinct subset called Cancer Stem Cells (CSCs). Another advantage of this model is the maintenance of CSCs. These cells have been purified and characterized by several types of PDX tumors through specific surface markers, which can be used for drug screening and discovery studies [43].

### Co-clinical trials

PDX models have accelerated the development of medications and the phases of clinical trials. Currently, the prediction value of PDX models is used in co-clinical trials. Phases 1/2 of the clinical trials take more than 5 years. Due to limitations in analysis and data integration, co-clinical trials have been suggested at this time (mouse hospitals are manifested), whereby new drug therapies are performed on experimental tumor models concurrently with clinical trials. Finally, the pre-clinical and clinical data are aggregated [44, 45]. The approach of the co-clinical trial using the PDX model facilitates the prioritization of optimal medications, facilitates rapid classification of respondents, determines biomarkers, and detects resistance mechanisms [44]. This format of trials has been or is being conducted for some cancers, such as Sarcoma [46], and Solid tumors [47] (Fig. 4B).

### Personalized medicine

PDX enables the growth of a tumor from any patient in an in vivo system called “Avatar models” or “mirror model”. Avatar is mainly used in personalized medicine approaches. They use the predictive capacity of this model for a particular patient. This model allows for the evaluation of drug sensitivity and precision drug efficacy and eliminates the cost of non-targeted therapy by providing a personalized regimen [48]. For example, Kopetz et al. established an Avatar model of colorectal cancer with BRAF mutation, which by using vemurafenib as an oral BRAF inhibitor, drug resistance was observed due to KRAS (Kirsten Rat Sarcoma Virus) and NRAS (Neuroblastoma RAS Viral Oncogene Homolog) mutations at low allele frequency in this tumor subset [49]. Each patient can have an equivalent animal, assuming that the animal model (PDX) has a similar function in the patient. Additionally, the acquisition of tumor profiles at different times with different therapies by PDX models allows for the evaluation and understanding of various molecular factors, signaling pathways, the flow of tumor growth metabolism, and molecular changes leading to metastases and drug resistance [48].

### Immunotherapy

In recent years, immunotherapy has achieved widespread success against various malignancies, so the limitations of the xenograft cell line-based model and Genetically Engineered Mouse Model (GEMM) have led researchers to become more inclined towards PDX models by reconstitution human immunity in PDX models and to create humanized models, scientists will be able to evaluate immunotherapies. In this approach, the correlation between the hematopoietic system and the tumor of the same patient is necessary. Humanized PDX models by reconstituting the human immune system and tumor growth enable the investigator to study tumor biology and immune system functionality. Zhao et al. have been developed humanized PDX model by type I human leukocyte antigen matched human immune system in NOD-SCID Il2rg−/− (NSG) mice as an immuno-oncology model to study immunotherapy approaches which both therapeutic and side effects of pembrolizumab and ipilimumab had been investigated in this model [50]. Immune check point investigation in gastric cancer humanized avatar model by engrafting the human tumor and PBMC injection showed delayed tumor growth by using anti-check point monoclonal antibodies [51]. These Humanized models have been used to evaluate many targeted therapies such as anti-PD1/PDL-1, CTLA-4, and other checkpoint inhibitors [52–54].

**Fig. 5:**
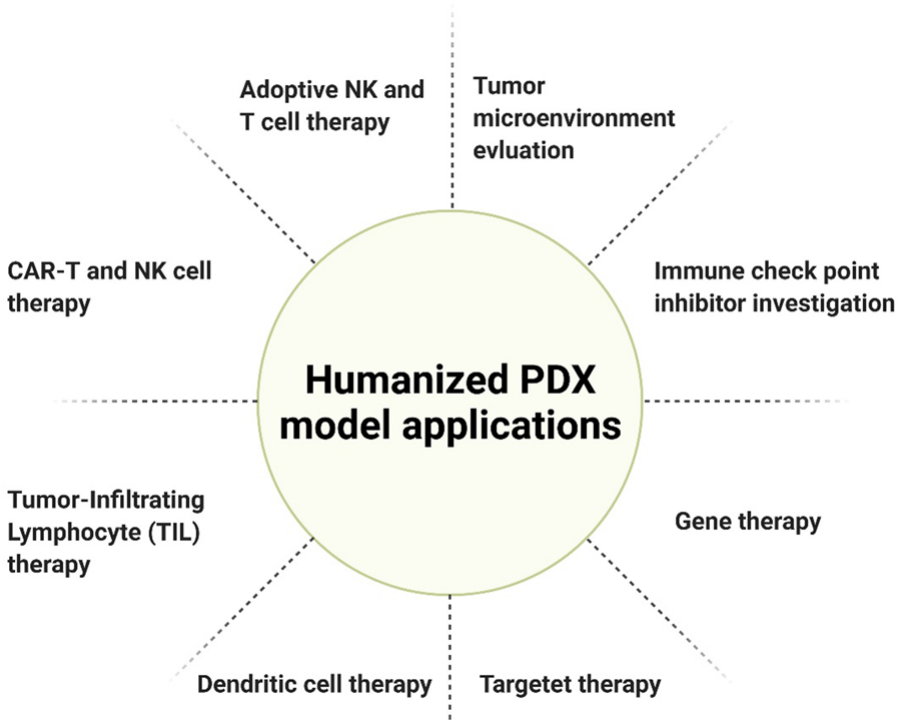
Humanized PDX model applications in immunotherapy

In 2010, the autologous CAR-T effect on PDX models of the same patient was evaluated for the first time [55]. In another study, PDX models of three hepatocellular carcinoma patients were developed, all of which retained the primary tumor characteristics, CAR-T inhibited tumor growth in the first group, and the tumor was eliminated in the other two groups [56]. In a 2017 study, the pancreatic cancer PDX models were used to evaluate anti-mesothelin CAR-T cells, which effectively inhibited the tumor [57]. Another application of PDX models in cell therapy is assessing various aspects of CAR-T cell treatment and biology. One of these aspects is the interaction between CAR-T and other immune cells such as Tregs and Myeloid-Derived Suppressor Cells (MDSCs) in the tumor microenvironment. These immune-inhibiting cells could be one of the causes of the negative results of some CAR-T trials. In the presence of these immune-inhibiting cells, PDX models will show more accurate and acceptable results, eventually, models with Patient immune systems such as humanized PDX models have received more attention from immunotherapy researchers [58]. Humanized PDX models are future tools in personalized medicine and will support clinical decisions. In an avatar (hIL2-NOG mice) of human melanoma patients, anti-PD-1 (Programmed Cell death Protein 1) antibody response and tumor-infiltrating T cells supported the clinical decision for immunotherapy [59]. However, the humanized immune-PDX models still need to be more validated. Still, they can eventually be a suitable approach for personalized medicine and clinical decision, especially for novel cancer immunotherapies (Fig. 5).

In xenograft models used in immunotherapy, the type of immunodeficient mice should be considered. As shown in Table 4, NOD/SCID /IL2rγnull (NSG) mice suitable for immunotherapy, these mice lack the production of cytokines such as IL-2, IL-4, IL-7, IL-9, IL-15 and, IL-21, which cause the loss of immune cells B, T, and NK, so these rodents are essential for transplantation of primary human tumors, while other immunocompromised mice are suitable for the development of cancer cell line-derived models [60, 61].

**Table 4:** Types of immunocompromised mice that can be used to develop cancer models

Mice generations	Immune defections	Features	Ref.
Athymic nude mice	Homozygous mutations in the Foxn1 gene (encoding the Forkhead box N1 transcription factor)	Defects in hair follicles and more importantly in the thymus and cause cells of B and T to lose their function. But antigen-presenting cells (APCs), macrophages, and NK cells are active in these mice	[19, 132]
Rag1/Rag2 mice	Rag1/2 recombinase defects	B and T cells in these mice lose normal performance due to the loss of function of the recombinase, such as athymic nude. These mice are particularly used to evaluate DNA damaging therapies because Rag knockout mice have a high tolerance for DNA damage	[19, 133]
SCID mice	Mutations in the Prkdc gene (protein kinase, DNA activated, catalytic polypeptide)	B and T cell leakage in these mice causes cellular immunity. Commonly, other mutations are used in combination with SCID mutations. The possibility of the death of these mice is very high due to spontaneous T-cell lymphomas	[134, 135]
SCID/Beige mice	Combination of Beige mutation (Bg) with the SCID mutation	To defects in B- and T-cells, Bg mutation causes defects in lysosomes and the function of NK cells, but on the other hand, this mutation causes a threefold increase in macrophages compared to the Wild Balb/C	[136, 137]
NOD/SCID mice	NOD (non-obese diabetic) mutation along with SCID	Directly affects cellular immunity by interfering with the performance of NK, APC, and macrophage cells	[138, 139]
NOG and NSG mice	NOD/SCID mice with IL2 receptor gamma truncation/disruption mutations	The incidence of lymphoma in NSG mice is lower than in NOD/SCID mice, and they have longer life spans, making them grow up to engraft more human tumors. These mice are suitable for the transplantation of low-growth tumors	[140, 141]
NRG mice	In NOG mice, Rag1 mutation replaced the SCID mutation	NRG has been used for intra-oral injection for PDX development	[140]

## PDX model achievements for five common cancers

The PDX model has been developed for various cancers over the years and has used for tumor evaluation and drug screening (Table 5). Here’s the application of this model is reviewed in five common cancers worldwide.

**Table 5:** Five common PDX models properties

Origin	Tumor take rate	Tumor latency	FDA approved protein biomarkers	Application of biomarkers	ECM (Matrigel)	Ref.
NSCLC	24%; 25/102	≥ 5 months	EGFR1, MMP7, CA6, KIT, CRP, C9, and SERPINA3 (not FDA approved)	Diagnosis	–	[28, 142–144]
Gastric cancer	15%; 35/232	3 months	Mast/stem cell growth factor receptor (SCFR)/c-Kit	Diagnosis, treatment selection	Yes	[145]
Colorectal cancer	63%; 54/85	2 months	Carcinoembryonic antigen (CEA), fibrin, fibrinogen degradation product DR-70	Disease monitoring, treatment response, progression Disease monitoring, diagnostics	Yes	[73, 76]
Breast cancer	15%; 20/130	4–5 months	CA 27, 29-CA 15-3 Estrogen receptor (ER), progesterone receptor (PgR), human epidermal growth factor receptor 2 (HER/Neu)	Monitoring disease, treatment response Prognosis, treatment selection	–	[146, 147]
Prostate cancer	10–20%; 21/261	4–12 months	Prostate-specific antigen (PSA) and p63	Disease monitoring, diagnostics Differential diagnosis	–	[87, 146]

### Lung cancer

PDX of Non-Small-Cell Lung Carcinoma (NSCLC) by imitating the molecular pattern of mutations and histopathological characteristics had been reported on 127 stable models by Wang et al. [62]. In the following, they showed that these emulator models are as same as their donors in gene expression profiling [62]. One hundred surgically resected specimens had been used to develop the PDX model of NSCLC by Ilie and colleagues. Among them, 35 models were established, and anything the team expects from these models was met, similar to tumor specimen [63]. These models have been used to evaluate common drug resistance such as docetaxel, cisplatin and can also be used to identify therapeutic predictive biomarkers [28, 64]. Lung tumor tissue samples were implanted under the sub-renal capsule in immunodeficient mice at a ˃ 95% rate [65].

Lung cancer patients who are at greater risk of recurrence after surgery are predictable, Due to the success of the PDX model’s engraftment rate. Similarly, in a study of 63 of 157 tissue samples of lung cancer transplanted to mice, they were compared with the clinical outcomes of patients, which were predicted to have a lower survival rate than others in a multivariate analysis [25].

### Breast cancer

PDX models provide a robust basis to study this fertile soil. First, cancer-related female death is not a single disease but a network of mutations’ which may lead to the development of new target therapies that, in turn, a preclinical in vivo model is needed [66]. The first transplantation of mammary tumor lines had been established in 1903, till 2003 that the efficient engraftment of human tumor tissue had been done in immunodeficient mice, CDX models of HER2+ (Epidermal growth factor Receptor 2) models, GEMM model of the BRCA1 (Breast Cancer Gene 1) mutation, and spontaneous mammary adenocarcinoma in transgenic mice were the most available in vivo models to study targeted therapies [67].

Initially, models of hormone-dependent cancers had many challenges. But over time and further research into the orthotopic transplantation of breast cancer tissue in the mammary fat pads of immunodeficient mice, estrogen supplementation has achieved some favorable engraftment rates [68, 69] (Table 2). The different parts of tumor tissue had been reported to use for Xenograft model establishment; primary breast, pleural effusion, ascites, and metastatic sites like the ovarian, brain, Nodal, skin, and soft tissue. Statistically Controversial metastatic site was found between the PDX model and human tumor; bone and brain metastasis are more frequent in patients but lymph nodes and lung in PDXs [18]. Triple-negative (ER-negative, PR-negative, and HER2-negative) tumor tissues have higher engraftment rates due to the aggressiveness of this type of cancer [69].

### Colorectal cancer

Various studies have shown that PDX models maintain heterogeneity of the Colorectal Cancer (CRC) primary tumor [70, 71]. The results of drug evaluations are most similar to the clinical settings. The rate of engraftment is dependent on the immunodeficient mice strain and the method of delivery. Tumor tissue is usually transplanted heterotopically into the subcutaneous or sub-renal site [72, 73]. However, several studies have isolated tumor tissue cells by enzymatic digestion and used them to develop this model [74, 75]. In other research, orthotopically, scientists developed CRC PDX models with endogenous metastases that can migrate to the lungs and liver similar to a patient’s primary tumor. Subcutaneous engraftment does not metastasize to other organs [74, 76, 77]. PDX models with NOD/SCID mice have the highest engraftment rate in this cancer [9]. PDX CRC models maintain important gene mutations such as KRAS, BRAF (v-Raf murine sarcoma viral oncogene homolog B), PIK3CA (Phosphatidylinositol-4, 5-bisphosphate 3-kinase), gene expression, copy number changes, and microsatellite instability of the primary tumor [78, 79]. Almost all studies on preserving the stromal structure, histological differentiation, and histopathological subtypes by these models are in agreement [74, 76]. PDX model of colorectal cancer in the evaluation of response rates of drugs such as cetuximab and other systemic chemotherapeutic agents [76, 79, 80] as well as the cell line production [81, 82], drug and biomarker discovery [83, 84], further understanding of tumor biology [83] and colosphere production [85] have had broad applications.

### Prostate cancer

Prostate cancer PDX models have been introduced as models with molecular diversity and cellular heterogeneity that have similar histology compared to primary tumors [86, 87]. Prostate cancer PDX models can be used to evaluate the efficacy of anti-cancer drugs [88]. However, the transplantation rate of human prostate cancer was low, and only advanced and high-growth cancerous tissue was successfully transplanted [89]. The sub renal capsule (SRC) site has a high transplantation rate because it has highly vascularized potential than the subcutaneous site for primary human prostate cancer; however, orthotropic models have shown the best expression of androgen receptor and Prostate specific antigen (PSA) [90, 91]. Another approach recently considered to increase the rate of transplantation is the use of CTCs, which have been used to produce various cancers, including the PDX prostate model. In this type of PDX model, there is no need for surgery to remove the tumor tissue [92]. The prostate tumor take rate is low 10–20% and requires Supplementing mice with a specific dose of exogenous androgens [87]. Different PDX models of prostate cancer for performing pre-clinical studies and evaluating biological processes such as identification of pluripotent stem cells [93], angiogenesis [94], response to androgen ablation therapies [95], and Chromosomal abnormalities [96] have been created over the years.

### Gastric cancer

Gastric cancer cell lines and animal models derived cell lines were used for drug screening; although these models had advantages, most importantly, the low predictive value of these models for clinical outcomes makes them inappropriate [97, 98]. The primary way to overcome these limitations is to use PDX that allows the transplantation of a patient’s tumor tissue into immunocompromised mice to form a population of mice with a tumor of the same molecular pattern. Suitable for evaluating targeted and personalized therapies [37, 80]. The most important achievement of an ideal preclinical model is preserving the genetic and histological features of the patient’s tumor, which are kept in the PDX model of gastric cancer for several generations [99–101]. Various factors have contributed to increasing the success rate of PDX modeling, including engraftment site [28, 37] using Matrigel [102] and type of immunocompromised mice [37, 103]. However, the diffuse type of gastric cancer is reported to have a low engraftment rate due to insufficient tumor cells [104]. Epstein bar virus (EBV)-related B-cell lymphomas infect approximately 68% of PDX tumor models [102, 105]. These are due to the presence of EBV in the patient's primary transplanted B cells, which are activated in the body of immunocompromised mice and cause lymphoma instead of the tumor [106]. PDX models have helped to improve gastric cancer therapies in recent years. Because of their in vivo benefits, and have been used in numerous pre-clinical trials to evaluate treatment response and identify biomarkers [38].

## Any challenges?

It seems animal models are still more available and also reliable with known challenges. Avatar or PDX models have high predictive power and are widely used in drug screening. Still, the long-time tumor latency (4–6 months) is perhaps the most prominent weakness from a personalized medicine perspective. Another limitation of these models is the different take rates in various cancers and hosts. For example, as discussed above, developing breast cancer models is more challenging than other cancers [17, 107]. PDX models are a valuable tool in oncology research and drug evaluation, but they are costly and require equipped laboratories. Another challenge we face in these models is to create an acceptable metastatic model. Usually, two approaches are used to develop metastasis. First, the tumor is grafted heterotopically or orthotopically, but in the second approach, patient-derived tumor cells are injected into mice through the tail vein. Both methods have limitations besides advantages. For example, in the first approach, spontaneous metastasis takes a long time. The rate of metastasis is low, on the other hand, by injecting tumor cells, despite the increase in metastasis rate. Still, we are dealing with an unrealistic heterogeneity, and in fact, most cells are trapped in the lungs rather than metastasizing to the lungs [108].

PDX models are also not suitable for early-stage cancers because of their low take rate. Due to the host with immunodeficiency, it is impossible to evaluate the effect of the immune system on the tumor [22]. This limitation makes it impossible to assess the relationship between the therapeutics cells and the host immune cells (such as Tregs and MDSCs) in cell-based therapies [60]. However, with the advent of Humanized models, this limitation has been partially removed.

Furthermore, there are epigenetic alterations of tumors among hierarchical generations, new mutations, and angiogenesis system of the host, which feds the esurient tumor cells are limitations. As Todd Glub, head of the cancer program at the Broad Institute, and his colleagues analyzed, the median of 12% of the genome had been affected by the fourth passage [109]. Of course, genetic alterations or chromosome abrasions are inevitable, but more efforts should be made to investigate how much these alterations are functional and will affect the outcome. However, the PDX model is essential option for tumor simulation. These limitations can be overcome by introducing a new generation of immunodeficient mice or efficient methods such as orthotopic cancer models and Humanized mice; humanized mice are models with little or without immune system which are injected with human stem cells derived from cord blood or fetal tissue. In these models human immune system included T cells, B cells and other cells will be created and enables scientist to explore human immune system function directly, but again the angiogenesis system will remain unresolved; as the vascular tissues of the tumor will be replaced by mouse angiogenesis system gradually during different passages [110]. Patient-derived tumor organoids (PDTOs) have been suggested as preclinical models that demonstrated higher success rate and ease of use, however they will not be able to preserve tumor heterogeneity. It seems more opportunities will be obtained in precision oncology era by integration of PDX and PDTO models [111].

When using PDX models, it should be noted that a transplanted tumor can trigger an immune response against the host that is called Graft Versus Host Diseases (GVHD), which can be delayed by using NSG mice without Major Histocompatibility Complex-I (MHC-I) [71, 112]. Another challenge that can be mentioned is Tumor Infiltration Lymphocyte (TILs) in the primary tumor, which may transform the tumor into lymphoma [113]. Post-Transplant Lymphoproliferative Disorder (PTLD) can be another challenge that may arise in creating PDX models. Choi et al., showed that NOG mice had a higher rate of EBV-related B-cell lymphoma development owing to their immunodeficiency. In contrast, lymphoma did not survive in nude mice, which could be due to Natural Killer cells in these mice [114]. If tumor tissue is transplanted to nude mice in the first generation, we will not have lymphoma in subsequent generations, even if using mice with maximal immune deficiency such as NOG, NSG [115, 116]. Rituximab can also be used as a treatment for lymphoma to inhibit its growth in PDX models without affecting the rate of cancer engraftment [36].

In recent years, many attempts have been made to provide in vitro models, including organoids and microfluidics, which will reduce the use of animals according to the principle of reduction from Replacement, Reduction, and Refinement (3R) rules. However, they have a long way to go to become a reliable model. PDX model centers are required to heed the notices and rules of ethics committees and animal rights organizations. They have introduced laws and regulations, which will not impede the development of PDX models but it will bring pacification to the animals.

## Conclusion

PDX models are among the most reliable and standard models in preclinical studies about a century after the first tumor model. Although these models, like other models, have limitations, they have many applications in the development of anti-cancer drugs, cancer biology, co-clinical trials, personalized medicine, and immunotherapy because of maintaining primary tumor characteristics. Therefore, many countries and institutes have begun to create PDX biobanks, and PDX libraries and pharmaceutical companies spend a lot of money to produce this source.

## Data Availability

All data generated or analysed during this study are included in this published article.

## Abbreviations

PDX: Patient derived xenograft
TECs: Tumor endothelial cells
CAFs: Cancer-associated fibroblasts
TAMs: Tumor-associated macrophages
ECM: Extra Cellular Matrix
FBS: Fetal bovine serum
PCTs: PDX clinical trials
CSCs: Cancer stem cells
GEMM: Genetically engineered mouse model
MDSCs: Myeloid-derived suppressor cells
PD-1: Programmed cell death protein 1
PD-L1: Programmed death-ligand 1
CTLA4: Cytotoxic T-lymphocyte-associated protein 4
CAR-T: Chimeric antigen receptor
NSCLC: Non-small-cell lung carcinoma
Her2: Epidermal growth factor receptor 2
CRC: Colorectal cancer
KRAS: Kirsten rat sarcoma virus
BRAF: V-Raf murine sarcoma viral oncogene homolog B
PIK3CA: Phosphatidylinositol-4, 5-bisphosphate 3-kinase
SRC: The sub renal capsule
CTC: Circulating tumor cells
PSA: Prostate specific antigen
MHC: Major histocompatibility complex
EBV: Epstein bar virus
GVHD: Graft versus host diseases
TILs: Tumor infiltration lymphocyte
PTLD: Post-transplant lymphoproliferative disorder
3Rs: Replacement, Reduction, and Refinement
